# Acute and transitional care or rehabilitation? Retrospective analysis of discharge planning from a municipal hospital in Switzerland

**DOI:** 10.1186/s12913-020-05547-1

**Published:** 2020-08-03

**Authors:** Lara Kollbrunner, Michael Rost, Insa Koné, Bettina Zimmermann, Yvonne Padrutt, Tenzin Wangmo, Bernice Elger

**Affiliations:** 1 Institute for Biomedical Ethics, University of Basel, Bernoullistrasse 28, 4056 Basel, Switzerland; 2 Institute of legal science, University of Zurich, Treichlerstrasse 10, 8032 Zürich, Switzerland; 3 Center for legal medicine, University of Geneva, Rue Michel-Servet 1, 1211 Geneva, Switzerland

**Keywords:** Discharge planning, DRG, Elderly patients, ATC, Rehabilitation, Switzerland, Retrospective data analysis

## Abstract

**Background:**

Due to rising health care costs, in 2012 Switzerland introduced SwissDRG, a reimbursement system for hospitals based on lump sum per case. To circumvent possible negative consequences like reduction in length of stay, acute and transitional care (ATC) was anchored into the law (Federal act on health insurance) in 2011. ATC as a discharge option is applicable to patients who physicians deem will not fulfill rehabilitation criteria, but are unable to return home and are in need of temporary professional nursing care. ATC is associated with higher out of pocket costs to the patient than rehabilitation. Since social service workers are responsible for organizing discharge for patients with ongoing care needs after hospitalization, the aim of this study was to investigate how social service workers manage patient discharge in light of the new discharge option ATC.

**Methods:**

Data was collected from 423 medical records of inpatients from Zurich’s municipal hospital, Triemli, discharged to ATC or rehabilitation, in 2016. We compared the two groups using inferential statistics and qualitatively analyzed written statements from social service workers.

**Results:**

Our results showed that patients discharged to rehabilitation had a higher total number of discussions, but a shorter duration of discussions. Patients discharged to rehabilitation faced more delays, mainly due to unavailability of beds in rehabilitation centers. Conflicts concerning discharge arose mainly because of costs, discharge placement and too early discharge.

**Conclusions:**

Our findings demonstrate how important social service workers are in providing information to patients about different discharge options. The newness of SwissDRG and ATC is still likely to cause longer discussion times and, consequently, more workload for social service workers. Only a small fraction of patients disagreed with their place of discharge, mostly due to financial reasons.

## Background

Switzerland has one of the best health care systems with good outcomes among the OECD-countries [1]. In relative terms, its health care system is the seventh most expensive in the world, consuming 10.9% of the gross national product [2, 3]. Since 1995, hospitalization costs were growing at a rate of 3% per annum, placing the question of health care costs and efficiency at the center of policy debates [4]. The Swiss Diagnosis Related Groups (SwissDRG) reimbursement system was introduced for hospitals to address rising health care costs [5]. It came into effect on 1st January 2012 and reimburses hospital services by lump sum per case mainly determined by diagnoses (taking into account comorbidities and other factors). The DRG payment system is generally expected to result in a reduction of length of stay (LOS) [6–8], raising concern that patients who need longer hospital stays or are in need of complex care might be discharged too early [9]. This vulnerable group is mostly presumed to be represented by elderly patients, children, patients with complex symptoms and patients without social network [10]. To circumvent the possible negative consequences of SwissDRG, readmissions within up to 18 days lead to no additional reimbursement for the hospital [11]. Furthermore, a new discharge option, Acute and Transitional Care (ATC) [12] was anchored into the Federal act on health insurance on 1st January 2011 as part of the nursing care financing system. This option was meant for those patients who do not require further hospital stay but still are in need of temporary professional nursing care and may be deemed unqualified for rehabilitation (hereafter Rehab). ATC is designed to support these patients in regaining their health and enabling their return home [13]. Although Rehab has a similar aim of ensuring that patients return to their independent state post injury, to receive this option several criteria must be fulfilled, such as patient’s deficits are clearly defined and recovery aims are feasible during the rehabilitation period [14]. Thus, physicians may deem a patient inappropriate for Rehab, when they are unable to ensure such clarity of needs and outcomes. How ATC is used, its financing and how it is understood by the patients remains unclear in Switzerland. However, ATC is organized variably in the different Swiss cantons (i.e. states) [15].

Unlike discharge to Rehab, where costs are covered by health insurance upon prior approval, discharge to ATC needs a medical prescription but no cost approval. ATC can be prescribed for a maximum of 14 days. The nursing care costs are covered by the health insurance and the canton, though costs for room and board are paid by the patients [5]. Thus, ATC has two important characteristics: it is associated with higher out of pocket costs to the patient and it does not require prior cost approval. It can therefore be much easier for physicians and social service workers to discharge patients to ATC than Rehab. It is feared that some patients, especially older patients who have capacity for Rehab might be discharged to ATC, where nursing care is the focus and fewer therapies are provided [13]. This is an undesirable outcome as intensive geriatric rehabilitation improves functional outcomes and reduces mortality [16]. Moreover, a published manuscript from the same project noted that ATC affects especially female patients because they are less likely to receive Rehab after hospitalization and that patients transferred to ATC probably receive less intensive therapies compared with patients discharged to Rehab [17].

In Switzerland, like elsewhere, social service workers organize the discharge of complex patients in close cooperation with medical personnel, patient and relatives [18, 19]. A study from the United States reported that social service worker are instrumental in resolving these complex combinations of discharge issues [20]. However, their roles remain unrecognized by hospital administrators, since they are typically not income generating [21]. A literature review on the effectiveness of hospital social service work concluded that hospital social service workers fail to express their value-added contributions to patients care and to clarify their role to other health care professionals [22].

This study sought to explore how social service workers managed patient discharge in light of the new discharge option, ATC, thereby comparing the new discharge option with the already existing Rehab option with its more rigorous admission criteria. By carrying out this comparison using the two discharge options available, we contribute to the previous works on this specific topic [17, 23]. Hence, this study examined the following questions:

I. What are the challenges social service workers face when attempting to triage hospitalized patients to the two post-acute care options: Rehab and ATC?
II. Do patients’ wishes influence discharge decision making?
III. What are the reasons for discharge disagreements and delays?

## Methods

This paper is part of a nationally funded project on ATC “Inpatient-outpatient transition in the era of DRGs: the legal framework and current practice” and presents findings from our analysis of social service workers’ notes retrieved from the medical records from Zurich’s municipal hospital, Triemli. The study was approved by the cantonal ethics committee Zurich (No 2015–0350).

### Data collection

The data for this cross-sectional study was collected from hospital records of inpatients who were 50 years or older, hospitalized during the period of April to August 2016 and for whom social service workers were involved in discharge management. This period of 5 months was chosen for resource reasons. The age limit was selected because older patients are likely to have a deficit in self-care, limited social resources and often need support post discharge [10, 15]. The study team collected data for 660 cases during the study period. For this analysis, we included data of patients released either to ATC or Rehab (*n* = 423; 10 patients contributed two records to the analysis).

From the electronic medical records, we extracted the following data: socio-demographic variables, social network of the patient (relatives, friends, neighbors etc.), living conditions, health related information (e.g. diagnosis, LOS, medication), discharge information and care needs related information. The extracted data included both numeric values as well as textual information. Data for this paper included all extracts where social service workers had documented their contacts with patients and third parties discussing care needs and options, number of discussions, total discussion time with patients/relatives, and patient information such as age, sex, martial status, place of discharge (ATC or Rehab) and LOS. In light of the retrospective nature of the study design, all this information were already existing routine information written down in the medical records. Thus, the social service workers (*N* = 14) did not receive any instruction from the research team on what data should be recorded during the study period.

### Data coding and analysis

To analyze the social service worker’s textual documentation, we prepared in advance a codebook based on our knowledge of data to capture quantitative information. This codebook was discussed with several researchers from the team. It included variables such as, if there was an explicit wish for Rehab or ATC from patient or third parties (i.e. family, friends, guardian, doctors), if there were disagreements concerning discharge (i.e. patient wants to be discharged to his or her home, but physician recommends ATC) or if there were delays (i.e. because of no free bed at the institution). These data were entered manually into IBM SPSS24 (SPSS Inc., Chicago, IL) and were double checked for correctness of data entry. There were no missing variables since no information was evaluated as “no”, except for mean delays in days. These quantified variables were analysed descriptively. Furthermore, we compared patients discharged to Rehab and patients discharged to ATC using independent samples t-test and Chi-square test of independence. Since extracted data pointed towards a higher number of conflicts for women as compared to men, we performed additional exploratory comparisons of these two groups. Due to the explorative nature of our analyses we did not adjust *p*-values for multiple testing [24]. For the reported analyses, *p-*values are 2-sided and statistical significance level was set at *p* ≤ .05.

In addition, the written statements in the medical records that contained information on discharge communications were coded using content analysis [25]. This qualitative analysis revealed explanations and reasons for the findings from the quantitative analysis. We analyzed the discussions between social service worker and patients, healthcare professionals or other parties to capture the reasons for disagreements between social service worker and patients and/or family members about discharge decisions, and what caused delays in arranging the discharge.

## Results

### Demographic characteristics of the sample

A small majority of our sample were women (57.7%). Age ranged from 50 to 99 years and length of hospital stay ranged from two to 73 days (Table 1). Further demographic characteristics for patients discharged to ATC, to Rehab, and the overall sample are presented in Table 1.

**Table 1:** Demographics and hospital stay

Variable	Mean (SD) / Percentage		
	ATC (*n* = 161)	Rehab (*n* = 262)	Overall (*n* = 423)
**Age** mean in years (SD)	82.91 (10.00)	73.92 (10.50)	77.34 (11.18)
**Gender** (% female)	68.3%	51.1%	57.7%
**Marital status** (% married)	28.6%	40.8%	36.2%
**Hospital stay** mean in days (SD)	13.00 (6.90)	16.86 (10.28)	15.40 (9.32)

### Discharge organization: ATC vs rehab

Information on number and duration of discharge communications, characteristics of these communications and incorporating patients’ wishes are presented in Table 2. Patients discharged to Rehab had a higher total number of discussions on discharge (*t* (421) = − 4.318, *p* = .000, d = .42); a higher number of discussions with third parties (*t* (421) = − 2.552, *p* = .011, d = .25); a shorter total duration of discussions (*t* (420) = − 2.224, *p* = .027, d = .22); and a higher number of third parties that were involved in the discussion (*t* (421) = − 2.937, *p* = .004, d = .30).

**Table 2:** Summary of comparisons

**a) Discharge organization**	**ATC** (*n* = 161)	**Rehab** (*n* = 262)
*Number and Duration of Communication on discharge between Social Worker and Patient*		
**Mean total of discussions with patients**^**a**^ (*n* = 423)	**2.0** (1.3)	**2.6** (1.57)
**Mean total of discussions with third parties**^**a**^ (*n* = 423)	**9.1** (5.64)	**10.6** (6.69)
**Mean total duration of discussions in minutes**^**a**^ (*n* = 423) ^1^	**141.7** (78.5)	**123.2** (86.1)
**Mean total of third parties involved**^**a**^ (*n* = 423)	**4.7** (1.2)	**5.1** (4.1)
*Characteristics of Communication on discharge between Social worker and Patient*		
**Informed about costs and possible providers**^**a**^ (*n* = 423)	**70.2%**	**80.5%**
**Documents were handed out**^**a**^ (*n* = 423)	**27.3%**	**54.6%**
**Possible solutions after hospitalization were discussed**^**a**^ (*n* = 423)	**47.8%**	**21.0%**
Patient was perceived to understand information (*n* = 423)	83.2%	90.1%
*Characteristics of Communication on discharge between Social worker and Patient*		
Disagreements (*n* = 423)	18.0%	12.2%
**Delayed discharge**^**a**^ (*n* = 423)	**17.4%**	**32.4%**
Mean delays (d) (*n* = 113)	6.5 (4.3)	5.8 (4.8)
**b) Gender**	**Men** (*n* = 179)	**Women** (*n* = 244)
**Age**^**a**^ (*n* = 423)	**74.7 (11.5)**	**79.3 (10.6)**
**Married**^**a**^ (*n* = 423)	**50.3%**	**25.8%**
**Living alone**^**a**^ (*n* = 423)	**27.4%**	**39.3%**
**Disagreement occurred**^**a**^ (*n* = 423)	**8.9%**	**18.4%**

Note. ^**a**^statistically significant; Standard deviation in parentheses; ^1^Because of the large standard deviation median and quartiles (which are insensitive to extreme values) are reported in the following. *ATC* Md*n* = 120, Q1 = 90, Q3 = 170, *Rehab* Md*n* = 100, Q1 = 65, Q3 = 150)

Moreover, patients discharged to Rehab had more discussions about costs and providers (*Χ*^*2*^ (1, *N* = 423) = 5.957, *p* = .015, *V* = .12); received informative documents more often (*Χ*^*2*^ (1, *N* = 423) = 30.025, *p* = .000, *V* = .27); and had less discussions about possible solutions (discharge options) after hospitalization (*Χ*^*2*^ (1, *N* = 423) = 33.447, *p* = .000, *V* = .28).

### Patients’ wishes and disagreement with discharge to ATC vs rehab

Seventy-three out of 75 patients who explicitly wanted to be discharged to Rehab were discharged to Rehab and 57 out of 60 patients who explicitly wanted to be discharged to ATC were discharged to ATC (data not presented). Among the patients that disagreed, the two patient groups (Table 2) did not differ with respect to the number of disagreements (*Χ*^*2*^ (1, *N* = 423) = 2.717, *p* = .0.99, *V* = .08). While more patients discharged to Rehab faced delays of discharge (32.4%) than patients discharged to ATC (17.4%) (*Χ*^*2*^ (1, *N* = 423) = 11.539, *p* = .001, *V* = .165), the mean delays (in days) for these two patient groups did not differ (*t* (111) = −.686, *p* = .461, d = .17). Disagreements regarding discharge possibilities occurred more often for women than for men (*Χ*^*2*^ (1, *N* = 423) = 7.557, *p* = .006, *V* = .134).

### Reasons for disagreements and delays

Table 3 presents reasons for disagreements, Table 4 reasons for delays. An exemplary extract from the data are presented in the tables for the reasons provided. Disagreements concerning discharge arose mainly because of discharge placement (57.4%), too early discharge (24.6%) and costs (9.8%). Main reasons for delayed discharges were medical issues (45.1%), unavailability of free places (32.7%), missing prior approval from insurance (10.6%) and social problems (5.3%).

**Table 3:** Examples of conflict evident from content analysis

Reasons for disagreement (*n* = 61)	Discharged to Rehab (*n* = 32)	Discharged to ATC (*n* = 29)
Costs (*n* = 6)	(*n* = 1) „Phone call from daughter: got 21 days neuro rehab in general department, daughter intervenes, that patient has semiprivate insurance. SW [social service worker]: [patient] is here in general department and was classified by patient admission like that. Daughter insists that patient goes to semiprivate rehab.”	(*n* = 5) „...furthermore, the husband consistently refers to the financial burdens of an ATC or the entrance in a nursing home, that would exceed the financial resources of the couple.”
Discharge Placement (*n* = 35)	(*n* = 18) „Discussion with patient: ATC discussed. Patient wants Rehab, Phone call from assistant physician: [Rehab] is clearly not indicated.”	(*n* = 17) „Discussion with patient: is feeling uncertain about going home and organizing everything alone again. Personal hygiene not independent at the moment and home care costs nearly the same like nursing home ➔ ATC, wherever.”
Discharge too early (*n* = 15)	(*n* = 10) „Phone call from son: is shocked about the early discharge.“	(*n* = 5) „Discussion with patient and husband: Patient is shocked that she has to go already. She doesn’t feel well at all.”
Two or more conflicts (*n* = 2)		(*n* = 2) „[Patient] doesn’t understand, that she couldn’t go home and instead needs ATC, because she can’t put weight on her leg. She is completely shocked about the high costs of ATC, because she doesn’t get any additional benefits.”
Other (*n* = 3)	(*n* = 3) „Exchange with nurses regarding transfer in private car; from the viewpoint of nurses, it is not possible. Patient absolutely wants to travel with a friend in private car. Clarification with physical therapist, same result. Discussion with patient: stubbornly refuses to give up.”

**Table 4:** Reasons for discharge delays

Reason for delay (*n* = 113)	Discharged to Rehab (*n* = 85)	Discharged to ATC (*n* = 28)
Missing cost approval (*n* = 12)	(*n* = 12) „Phone call from Rehab: they asked about the result of cost approval from insurance. Discharge will be delayed by Tuesday, when we will have the cost approval.”	
No free place (*n* = 37)	(*n* = 36) „Got cost approval for 14 days, but there is no free bed at the moment, only waiting list.”	(*n* = 1) „Phone call with assistant physician: information that unfortunately the patient’s discharge will be delayed by next Wednesday.”
Medical issues (*n* = 51)	(*n* = 33) „Phone call with assistant physician: discharge must be delayed by another day because of medical issues.”	(*n* = 18) „Patient felt bad, todays planned discharge is cancelled. According to the assistant physician, discharge is possible by the end of the week.”
Social problems (*n* = 6)	(*n* = 2) „Phone call with daughter: her daughter will be entering the same Rehab because of fatigue. The patient shouldn’t see her. We should, cancel Rehab without saying anything to patient. Phone call with assistance doctor: other Rehab has bed in 2.5 weeks, patient cannot stay till then.”	(*n* = 4) „It turns out, that ATC is necessary. Wife reports, that recently patient is angry and aggressive, home care doesn’t help. Wife needs protection. After consultation with assistant physician, discharge is delayed to Tuesday.”
Other (*n* = 6)	(*n* = 2) „Phone call from nurse: husband phoned Rehab, now they have a bed [by date], husband says that patient could stay till then. Phone call from assistant physician: no, that was never discussed like that, from medical point of view, patient is ready for discharge.”	(*n* = 4) „Discussion with wife and patient in the afternoon: for both the discharge tomorrow is too early. Got assistant physician to talk [with patient and wife]: from medical point of view, discharge tomorrow is fine. Compromise: day after tomorrow.”
Two or more (*n* = 1)		(*n* = 1) „Son puts pressure on Patient and daughter and uses violence. Discussion with patient, son and daughter: all agree that patient couldn’t go home, ATC is needed … According to nurse, patient fell during night and is now under permanent watch. Discharge uncertain.”

## Discussion

This study presents unique data revealing discharge planning after the introduction of SwissDRG and ATC. It highlights how social service workers use the new option of ATC in comparison with Rehab, and provides details about disagreements that occur when planning discharge for patients who cannot live independently at home.

Concerning discharge organization, our study findings first underline that social service workers spent more time discussing discharge for patients sent to ATC than for Rehab-group, despite ATC-group’s shorter stay at the hospital, and a smaller number of discussions with patients and third parties. The longer total duration of discharge discussions with ATC patients could be explained by the fact that the latter was introduced only in 2011 and is yet to become well-known among patients [5]. Hence, social service workers take more time explaining this option. Long discussions about ATC can also be explained by the higher financial burden on the patient when this discharge option is used [13]. These long discussions with social service workers might be an additional burden in light of the fact that this patient group is likely to be older and possibly suffer from more severe diseases and comorbidities as compared to patients discharged to Rehab [26]. Furthermore, older patients may have (recognized or unrecognized) underlying cognitive impairments, which may contribute to difficulty with comprehension.

That women were more likely to have disagreements than men could be because they were more likely to be discharged to ATC [17], were older, and more often lived alone. Prior studies on ATC showed that there is a risk that ATC is allocated to vulnerable groups (i.e. elderly patients) [9]. Patients (and their families) might have a double interest in asking for Rehab: they pay less for it and because there is a waiting time, patients remain in the hospital for a longer period. Those who are unaware of this fact may not insist on Rehab and thus may have a higher chance of being discharged to ATC. Since physicians first decide whether or not a patient has potential for Rehab and the fact that they do so without clear guidelines it is a hurdle to make a Rehab discharge decision. Furthermore, the fact that their recommendation will require confirmation and approval from the health insurance is an additional barrier. These two hurdles combined with patients and families actively requesting (or not) Rehab are barriers and biases that may result in inconsistent decision-making regarding discharge. These findings highlight the important role that family caregivers play in discharge discussions and outcomes.

Our analysis further showed that social service workers considered the explicit wishes of patients and third parties for Rehab or ATC choices in most cases. This finding is important for patients as it underlines the significance of voicing their preferences. However, disagreements did take place and mostly occurred because patients wanted to be sent to a different place (some due to financial reasons) or because they perceived discharge to be too early. Fortunately, in all these cases, social workers were able to reach an agreement with the patients. These results reveal how crucial social service worker are in resolving these complex combinations of discharge issues [20].

Finally, our data show that Rehab-patients faced almost twice as many delays as ATC-patients, with the main reason being unavailability of a place at Rehab institutions. Hence, patients discharged to Rehab stayed longer in the hospital than patients discharged to ATC since immediate prior hospitalization is one of the criteria to receive cost approval for Rehab [27]. Consequently, patients unnecessarily stay in the hospital until a place is available in rehabilitation centers, which counters the goal of SwissDRG and possibly results in financial loss for the hospital [15]. In addition, prolonged hospitalization increases their risk for in-hospital complications [28–30], thereby questioning whether regulations must be adapted to address real world situations and to ensure that they do not interfere with the best interest of patients.

### Future research

Further research is necessary to understand outcomes achieved by discharging patients to ATC compared to Rehab in terms of promoting return to independent living. Such outcomes could include for example, level of independency achieved, health status, readmission to hospital and associated levels of family caregiver burden. Also, the conflicts that arose, as shown in Table 3, bring out the important role of early preparation of patients and families for likely discharge scenarios. Thus, discharge preparation should begin in a timely fashion. Technological innovations such as professionally developed videos that provide information in a consistent manner, could help social service workers to explain these difficult tasks to the patients in a more standardized way. Since discharge decisions are prone to biases, hospitals should train its physicians and social workers in deciding how to allocate patients to the best discharge option.

## Limitations

Our analysis is based on written social service worker’s notes in medical records and therefore represents a single perspective. Furthermore, since this information are noted post-hoc by the social workers, it represents only a distillation of what they recalled from their discussions with the patients. Our findings do not encompass important and direct input from patients and families. However, another component of the project uses qualitative interviews with patients sought to capture patient experiences [31]. We cannot guarantee that the data extracted from medical records are complete since information not written down in medical records could have been given to the patients. We only collected data at one hospital for a period of 5 months due to financial and time constraints. Thus, caution is necessary with respect to interpreting our data and its generalizability. Finally, the adaptation of ATC in Switzerland is very heterogeneous in different regions (cantons) and, as noted before, details of cantonal practices are not well known [15].

## Conclusions

Our findings reveal that social service workers provide intensive support with respect to organization of discharge. Our results are a strong message for policy-makers to re-evaluate existing regulations to prevent risk selection or insufficient treatment of vulnerable groups [32]. Finally, study results demonstrate that the newness of SwissDRG and ATC is still likely to cause longer discussion times and, consequently, more workload for social service workers. This also shows how important social service workers are in explaining to and discussing with patients about old and new discharge options.

## Data Availability

The datasets used and/or analysed during the current study are available from the corresponding author on reasonable request.

## Abbreviations

ATC: Acute and Transitional Care
LOS: Length of hospital stay
Rehab: Rehabilitation
SwissDRG: Diagnosis-Related Groups in Switzerland
